# Adenovirus-Derived Nano-Capsid Platforms for Targeted Delivery and Penetration of Macromolecules into Resistant and Metastatic Tumors

**DOI:** 10.3390/cancers15123240

**Published:** 2023-06-19

**Authors:** Rebecca Leah Benhaghnazar, Lali Medina-Kauwe

**Affiliations:** 1 Department of Biomedical Sciences, Cedars-Sinai Medical Center, Los Angeles, CA 90048, USA; medinal@cshs.org; 2 Geffen School of Medicine, University of California, Los Angeles, CA 90095, USA

**Keywords:** macromolecule, macromolecular delivery, adenovirus penton base, penton base, biological therapeutics, tumor targeting, HER3, metastatic, resistant

## Abstract

**Simple Summary:**

Many cancer therapeutics may have a robust effect on target molecules but lack the means of effective localization into tumor cells, thus displaying reduced therapeutic efficacy. Viruses bear naturally evolved machinery to ensure entry and localization of nucleic acid cargo into a cell. Utilizing and better understanding the mechanism of the penton base protein that contributes to the adenovirus cell entry machinery may improve the intracellular delivery of tumor targeting therapeutics. This review describes the progression of penton-base-derived cancer nanotherapeutic development and highlights essential requirements for robust delivery into a tumor cell.

**Abstract:**

Macromolecular therapeutics such as nucleic acids, peptides, and proteins have the potential to overcome treatment barriers for cancer. For example, nucleic acid or peptide biologics may offer an alternative strategy for attacking otherwise undruggable therapeutic targets such as transcription factors and similar oncologic drivers. Delivery of biological therapeutics into tumor cells requires a robust system of cell penetration to access therapeutic targets within the cell interior. A highly effective means of accomplishing this may be borrowed from cell-penetrating pathogens such as viruses. In particular, the cell entry function of the adenovirus penton base capsid protein has been effective at penetrating tumor cells for the intracellular deposition of macromolecular therapies and membrane-impermeable drugs. Here, we provide an overview describing the evolution of tumor-targeted penton-base-derived nano-capsids as a framework for discussing the requirements for overcoming key barriers to macromolecular delivery. The development and pre-clinical testing of these proteins for therapeutic delivery has begun to also uncover the elusive mechanism underlying the membrane-penetrating function of the penton base. An understanding of this mechanism may unlock the potential for macromolecular therapeutics to be effectively delivered into cancer cells and to provide a treatment option for tumors resisting current clinical therapies.

## 1. Introduction

Cancer is a leading cause of premature death in the majority of countries worldwide [1]. Standard therapies include surgery, radiation, chemotherapy and antibody-based treatments [2]. Although there have been breakthroughs in the field of immunotherapy for cancer treatment in combination with conventional cancer therapies, there is still a dire clinical need to effectively target cancer cells to reduce off-target toxicity while preventing metastasis and recurrence [3]. Additionally, resistance mechanisms arising within the tumor cell may abrogate the efficacy of antibody or small molecule-based therapies [4,5,6], thus highlighting the need for alternative therapeutic approaches that may be addressed through the delivery of nucleic acids or other macromolecules [7,8]. Since the majority of molecular targets in the cell require therapeutic access to the cytoplasm, it is essential that these macromolecule therapies can target and penetrate the plasma membrane of cancer cells and can be delivered and localized at their intended destination within the cell. Accordingly, key biological barriers must be overcome to ensure that the macromolecule is efficiently internalized and can penetrate through the cell membrane to reach its intended therapeutic target.

Viruses bear highly evolved means of penetrating cell barriers for the targeted delivery of viral nucleic acid, and such functions could be beneficial for targeted macromolecular delivery into tumor tissue. Derivatives of the DNA virus, human adenovirus, have been explored as a means of cell entry for cancer therapeutic targeting and construct design [9]. In particular, the penton base of the adenovirus bears a robust cell entry mechanism that facilitates virus internalization and membrane penetration [10,11]. Accordingly, the penton base protein alone has been used to target and penetrate cancer cells for mediating cell entry and intracellular localization of therapeutics while avoiding the concerns associated with using the whole virus including infectivity, immunogenicity, and potential recombination with wild-type viruses [12,13,14,15,16,17,18,19]. Nanocapsid platforms utilizing the penton base, the penetration engine of the adenovirus, have shown the ability to penetrate resistant and metastatic tumor cells to deliver therapeutic macromolecules [20,21,22,23]. In particular, the penton-base-derived HerPBK10 (or HPK) nano-construct coalesces tumor homing, membrane penetration, and viral capsid-like cargo encapsulation functions within a single fusion protein for systemic therapeutic delivery to resistant and metastatic tumors [18]. HPK utilizes a tumor-homing strategy with applicability for a potentially broad range of resistant and metastatic tumor types. Accordingly, the development of HPK will serve as a unique point of reference for discussing essential functions needed in nano- or biological vehicles constructed for delivering macromolecular cargo.

In this review, we first provide an overview of cellular uptake barriers for macromolecular delivery and the adenovirus biology that has evolved to traverse such cellular barriers. We then focus on the functional domains integrated into HPK that are intended to mimic the early stages of adenovirus infection (cell binding, entry, and endosomolysis) that can benefit macromolecular therapeutic delivery. Finally, we discuss how these functions may be leveraged for advancing development of targeted therapies to address resistant and metastatic tumors.

## 2. Requirements for Macromolecular Cell Entry

**Figure 1:**
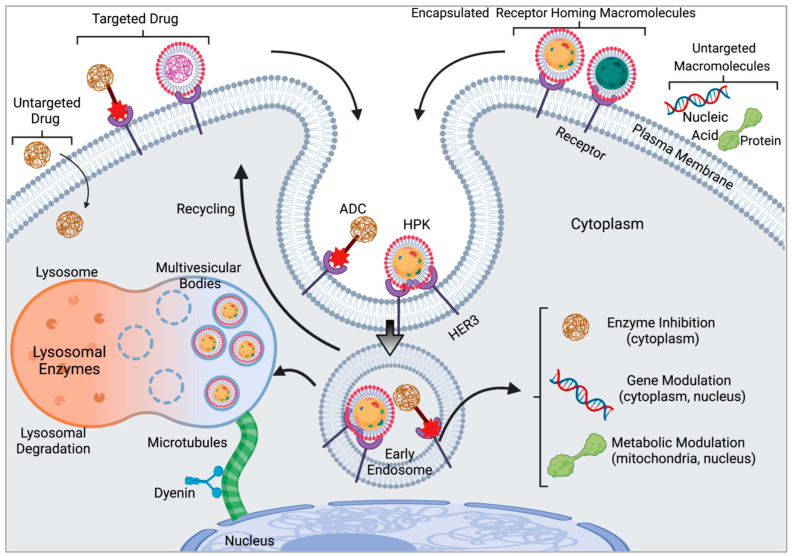
Subcellular barriers to macromolecular delivery and HPK utilization of the endosomolytic pathway. Untargeted drugs that are membrane permeable can readily enter cells but require an endocytic pathway of uptake if tethered to a targeting ligand. Likewise, macromolecules including nucleic acids and proteins are generally unable to traverse the plasma membrane without an endocytic uptake process that includes lysis of the endosomal membrane (endosomolysis). Receptor homing bioparticles and targeted drugs including antibody drug conjugates (ADCs) require entry through receptor-mediated endocytosis but can also recycle out of the of the cell (indicated with arrow). The targeted penton base construct HPK binds HER3 and enters the early endosome. The HPK capsomere can disassociate into monomers in response to low pH (which typifies a maturing endosomal vesicle) that may enable evasion of lysosomal degradation, then migrate toward the nucleus by way of microtubules (green) via the cytoplasmic dynein. Schematic not drawn to scale. Image created using BioRender.

Many small molecules and drugs such as the chemotherapy agent, doxorubicin, can readily diffuse across the cell membrane and thus enter many different cell types; but once the molecule is attached to a targeting ligand or delivered through a ligand-targeted vehicle, cell entry is most likely directed through receptor-mediated endocytosis (Figure 1) [24]. Likewise, macromolecules such as nucleic acids, proteins and peptides are unable to breach the cell membrane on their own, and thus need to use endocytic pathways to enter the cell [25]. Different forms of endocytic uptake can mediate cell entry of macromolecular therapeutics including clathrin-mediated endocytosis, caveolar/raft-dependent endocytosis, and clathrin–caveolin/raft-independent pathways such as phagocytosis and pinocytosis [26,27]. In each of these cases, endocytic uptake results in the formation of vesicles that bud inward from the cell membrane, thus encapsulating the endocytosed cargo in a cellular endosome (Figure 1). Early endosomes formed from clathrin-mediated endocytosis mature (through endosome acidification) to late endosomes that fuse with lysosomes to digest the endosomal contents (Figure 1) [28]. To escape this fate and to access therapeutic targets that are most likely extra-endosomal, a means of breaching the endosomal membrane is necessary (Figure 1) [26,29]. The time frame of endosomal escape is essential in determining whether the endocytosed matter is delivered to the desired location within the cell or whether it is degraded in lysosomes or recycled out of the cell (Figure 1) [30]. The use of viral particles can facilitate successful endosomal escape so that the contents of the endosome can be delivered to the cell cytoplasm and/or other extra-vesicular compartments to access therapeutic targets.

## 3. The Adenovirus Capsid as a Macromolecular Delivery Vehicle

Human adenoviruses, part of the mastadenovirus genera, are classified into seven different species or subgroups categorized from A through G [12,15]. In total, there are over eighty-five different serotypes within the seven species of adenoviruses, and the most studied serotypes include adenovirus serotype 2 (Ad2) and Ad5 of species C and Ad3 and Ad7 of species B [20,31]. A concern with the efficacy of using adenoviral vectors is that within the general population, immunity exists against adenoviruses, specifically the most common serotypes that would produce a humoral response once administered [32]. The most widely used as viral vectors are Species 2 and 5, primarily because humans are less commonly infected with those serotypes and therefore there is less pre-existing immunity [33,34].

### 3.1. Approved Adenovirus-Based Therapeutics for Treating Cancer and Other Indications

Half of all viral vectors used in clinical trials are adenovirus viral vectors [35]. The 36 kb Ad5 genome is comprises both “early” regions expressed before the viral DNA replication and “late” regions which are expressed after viral replication [36]. The early regions are not essential for transgene packaging, delivery, and expression; thus, adenoviral vectors with the E1 region deleted can accommodate transgene insertions of up to ~4.5 kb in size without exceeding the packaging capacity of the capsid and are therefore often used in transgene delivery [36]. Accordingly, gene therapy vectors such as Gendicine comprise a recombinant human Ad5 vector driving a transgene expression cassette inserted in the Early region 1 (E1) [37]. Gendicine delivers an exogenous gene expressing p53 and is approved by the State Food and Drug Administration (SFDA) of China in 2013 to treat squamous cell carcinoma of the head and neck (HNSCC) in combination with chemotherapy, which has been used to treat over 30,000 patients [38,39,40,41].

The gutless adenovirus (GLAd), also called a helper-dependent adenovirus (HDAd), allows for even larger transgene inserts due to the removal of all endogenous viral genes resulting in packaging of only the transgene (and any necessary stuffer sequence) within the adenovirus capsid [42]. Gutless Ads retain functional capsids, thus sustaining effective cell binding, internalization, endosomolysis and transit to the nucleus which comprise the early stages of host cell infection [43,44]. Additionally, not only the removal of the viral genome allows ample room for large (up to 36 kb) transgene inserts, but also the elimination of viral gene sequences reduces the amplification of the host immune response [45,46]. GLAds have shown therapeutic potential for Huntington’s disease (HD) and Duchenne muscular dystrophy (DMD) [42] which both involve large gene sizes. Importantly, GLAds demonstrate that the capsid proteins are sufficient for effective cell entry, endosomolysis, and trafficking via the cytoskeleton, highlighting their potential value for non-viral means of therapeutic delivery.

### 3.2. Structure of the Adenovirus Capsid

**Figure 2:**
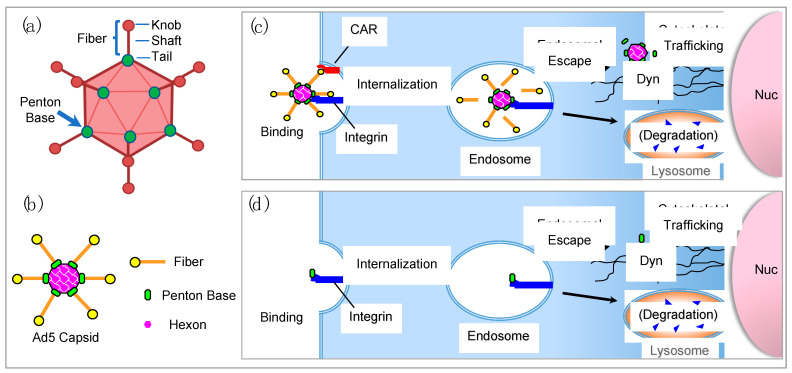
Adenovirus and soluble penton base cell entry. (**a**,**b**) Schematics of adenovirus serotype 5 (Ad5) capsid showing individual protein components. Schematics shown in (**b**) provide the symbol legend for schematics shown in (**c**,**d**). Not drawn to scale. (**c**) Schematic showing the early stages of Ad5 infection, delineating cell surface binding followed by internalization, endosomal escape, and dynein (Dyn)-mediated trafficking along microtubules toward the nucleus (Nuc). (**d**) Schematic of penton base recapitulating the trafficking pathway of whole Ad.

To understand the significance of the adenovirus capsid proteins in facilitating cell entry during the early stages of viral infection, it is useful to appreciate the structure and protein components comprising the viral capsid. The linear double-stranded DNA virus is 70 to 90 nm in diameter with an icosahedral shaped capsid. Adenoviral genomes vary in length from 26 kb to 45 kb depending on the serotype with inverse repeated sequences at each end and an encapsidation sequence [33]. The Ad2 or Ad5 virus capsid is composed of 252 proteins comprising three distinct protein types, including the hexon, the penton base, and the fiber (Figure 2a,b) [47,48].

Although the majority (80% by weight) of the viral capsid is composed of the hexon protein which comprises the polyhedral shell [15,49], it is the fiber and penton bases that initiate the early stages of infection.

The penton base is composed of a homopentamer residing at each of the 12 viral capsid vertices [50]. Each penton base contains hypervariable loops, including an arginine-glycine-aspartic acid (RGD) loop and a variable loop [50]. Binding with αvβ3 and αvβ5 integrins by the RGD motif facilitates internalization of many types of viruses [51], though some serotypes use alternate routes. For example, human adenovirus D serotype 40 (HAdV40) and human adenovirus F serotype 41 (HAdV41) lack the RGD motif but still successfully infect cells [52]. In particular, the penton bases of HAdV40 and HAdV41 can bind to α6-containing integrins with a binding affinity similar to that of penton base proteins containing the RGD motif that interact with alpha v integrins [53]. The penton base is conserved across serotypes and provides the same role of viral internalization in cells [10,54], hence imparting an essential conserved function in the virus life cycle.

The adenovirus fiber protrudes the likeness of an antenna from each penton base and consists of a homotrimer composed of three domains: the tail, shaft, and a knob domain. The fiber tail located at the amino [N]-terminus non-covalently binds the penton base unit. The receptor-binding knob domain is located at the carboxy [C]-terminal end of the fiber protein with a repeat sequence comprising the shaft separating the tail and the knob. The length of the shaft differs between serotypes depending on the number of repeated sequences of 15 amino acids [55,56].

### 3.3. Adenovirus Cell Entry

The Ad capsid mediates a two-step process for enabling entry into a cell (Figure 2c). The Coxsackievirus and Adenovirus Receptor (CAR) is the primary receptor that binds the knob domain of the adenovirus fiber of Serotypes 2 and 5 (as well as Serotypes 9, 12, and 41L) through an interaction occurring between the adjacent fiber knob monomers leading to receptor trimerization at the cell surface [33,57]. This interaction is followed by endocytic cell entry through the binding of integrins by the penton base [17]. The majority of human cells express both the primary adenovirus receptor—CAR—and the secondary integrin receptors, accounting in part for the broadly efficient infectivity of adenoviral vectors [58]. It is important to note that although CAR is the primary receptor for Ads, not all Ads engage this cell entry pathway; for example, the majority of the adenoviruses from subgroup D utilize glycan-bearing receptors for cell entry including GD1-glycan and sialic acid [59,60].

After cell surface binding, the virus enters the endocytic pathway triggered by the interaction of the penton base with the integrins inducing integrin clustering and the entry into endosomal vesicles (Figure 2c) [61]. Endosomal escape requires the release of capsid proteins including the penton base from the whole virus capsid, which in turn has been attributed to protein VI that tethers the capsid proteins to inner-capsid contents and releases the proteins in response to pH-responsive cleavage [62]. Protein VI, a 22 kDa protein underlying the peripentonal hexons of the adenoviral capsid, has been considered essential for the endosomolytic function of the whole virus as its mutant form, which prevents capsid dismantling and membrane lytic activity by the whole Ad [63,64]. As lysis of endosomal membranes requires disassembly of virion capsids [63], release of the penton base from the capsid would support the endosomolytic activity of soluble penton base.

Several corroborating studies show that soluble penton base protein can directly mediate disruption of the endosomal membrane, consistent with the need for the penton base to release from the virus capsid to effectuate endosomolysis [18,19,20,23]. The exact means by which the penton base disrupts the endosomal membrane integrity has long been unknown, although recent studies have begun to uncover this elusive mechanism as discussed later in this review. Studies on Ad5 intracellular trafficking show that post-endosomal capsids migrate toward the nucleus by way of the microtubules, specifically by interacting with the cytoplasmic dynein complexes that normally transport vesiculated cargo within the cell [65,66]. Importantly, soluble recombinant Ad5 penton base is capable of microtubule-mediated trafficking through the cytoplasm to reach the cell nucleus in the absence of the rest of the virus and in a similar manner to that of the whole adenovirus (Figure 2d) [23,61].

## 4. Penton Base-Derived Macromolecular Delivery by HerPBK10 (HPK)

**Figure 3:**
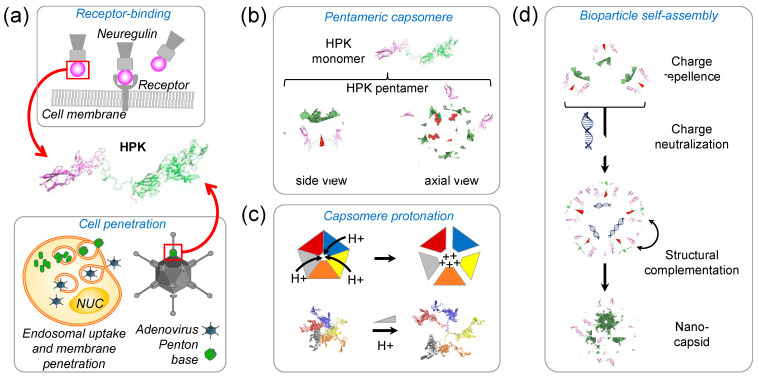
HPK monomer, pentamer, and nano-capsid assembly. (**a**) Schematic showing derivation of HPK fusion protein by combining gene sequences that encode the receptor-binding domain (magenta) of neuregulin fused to a penton base (green) sequence modified by a C-terminal decalysine (red). Figure shows ribbon structure of HPK monomer. (**b**) Schematic showing pentamerization of HPK in ribbon-structure format, with a view from an axial perspective, and side view. Functional domains are delineated by different colors: ligand binding (magenta); penton base (green); cargo binding (red). (**c**) Capsomere response to low pH. Upper figures, schematic depicting accumulation of proton ions (H+) in the center of capsomere barrels causing charge-mediated repellence of monomers from one another. Lower figures show molecular dynamics structural models of capsomeres responding in a low pH environment by exhibiting dispersion of monomers from one another. Each monomer is coded by a different color. (**d**) Cargo-triggered assembly of HPK into nano-capsids. HPK capsomeres shown in ribbon structure format with assembly sequence shown from top to bottom.

HerPBK10, a recombinant penton base chimeric protein derived from the adenovirus 5 (Ad5) serotype, was originally designed to employ the cell-penetrating activity of the penton base to deliver exogenous DNA for nonviral gene therapy applications and has since been evaluated for the transport of a variety of therapeutic payloads while targeting delivery to resistant and metastatic tumors [20,21,22,61,67]. Also known in its abbreviated form as HPK, with its earliest iteration created in 2001, HerPBK10 is composed of three essential parts comprising tumor targeting, membrane penetrating, and cargo loading functions (Figure 3a). The tumor targeting portion of HPK is derived from the binding ligand for HER3, specifically comprising the receptor binding portion of heregulin (or neuregulin) 1-⍺1; it also induces rapid receptor-mediated endocytosis (Figure 3a) [18,23]. The membrane-penetrating portion of HPK is derived from the penton base of Ad5 and facilitates endosomolysis or disruption of the endosomal membrane. The cargo-loading K10 domain of HPK allows for binding to an anionic payload. Each of the functional domains of HPK and the particular advantages they provide compared to their respective conventional functionalities are described in the sections below, starting with the foundational platform of HPK: the penton base.

### 4.1. The Penton Base as a Membrane Penetration Platform for HPK

The membrane-penetrating, or endosomolytic, portion of HPK is derived from the Ad5 penton base which also facilitates trafficking within the cell after endosomal escape [22,61,68]. These functions allow for subcellular delivery of macromolecule therapeutics which would be otherwise excluded by cellular and intracellular barriers [67,69].

The structure formed by the penton base reveals the putative mechanism of endosomolysis for both the wild-type penton base and HPK. Computational structural modeling based on the crystal structure of the recombinant soluble adenovirus penton base confirms that the protein in its natural form creates a homopentamer, or capsomere as found in its wild-type state at each viral capsid vertex (Figure 3b) [23,50]. Structural modeling of penton base and HPK capsomeres shows that the pentamer formation creates a barrel structure with a solvent-accessible inner pore lined with charged amino acids whose protonation under acidic conditions (such as the maturing endosome) causes repellence of monomers from one another and exposure of hydrophobic domains mediating pentamerization [23]. Specifically, histidines and negatively charged amino acids (glutamic acid and aspartic acid) counterbalanced by positively charged (lysine and arginine) amino acids line the capsomere inner pore (https://youtu.be/BB_hxCHbTMI, accessed on 2 March 2023) [23]. Protonation of the histidines and anionic residues in a low-pH environment induces the repellence of the charged monomers from one another, leading to capsomere disassociation (Figure 3c) and unmasking of hydrophobic domains mediating inter-monomer binding [23]. Hydrophobic domain interaction with endosomal membrane lipids may be sufficient for lipid destabilization in the endosomal membrane, as blocking acidification of maturing endosomes containing HPK prevents the escape of vesiculated fluorophore [61]. These findings corroborate earlier studies showing that soluble recombinant penton base can be seen escaping lysed endosomes under electron microscopy [70], can facilitate co-uptake and intracellular transcription of exogenous genes [61,70,71], can form homomeric dodecahedral structures that undergo post-endosomal trafficking toward the nucleus [23], and can mediate intracellular cytoplasmic delivery of membrane impermeable corrole compounds [21,67,68,72]. Microscopic and sub-cellular fractionation assays confirm that during cellular uptake, HPK shows overlap with early endosomes but not late endosomes or lysosomes, suggesting that HPK leaves the early endosomes after endocytosis [23,68].

### 4.2. Nano-Capsid Self-Assembly Nucleated by Therapeutic Cargo

The cargo-loading deca-lysine, or K10, domain of HPK allows for binding to an anionic payload by electrostatic interaction [18] (Figure 3d) while the structure of HPK capsomeres enables a unique mode of self-multimerization into bioparticle complexes. Specifically, the formation of capsomeres aligns the K10 domains of each monomer on the same façade of the capsomere barrel (Figure 3b), creating a highly charged cationic surface for repelling other HPK capsomeres in the solution while attracting anionic molecules [23]. Binding with anionic cargo such as small nucleic acids neutralizes the cationic façade of the capsomere barrel, allowing HPK capsomeres to converge around the cargo (Figure 3d). This convergence allows capsomeres to come into close molecular contact, enabling inter-capsomere motifs inherent to the penton base [23] to interact and fit together by shape complementarity (Figure 3d). This complementation combined with the cork-shaped structure imposed by the penton base nucleates the formation of a spherical polyhedron with the anionic cargo encapsulated by the protein shell (Figure 3d). Self-assembly of HPK capsomeres with either small nucleic acids, drug-intercalated nucleic acids, or small molecules forms similar-sized complexes of 20–40 nm in diameter whereby the HPK protein surrounds and encapsidates the cargo [22,23]. Functional studies have shown that this encapsulation enables protection of cargo from nucleases while sustaining stability in blood and serum [23]. On the other hand, the high stability afforded by the polyhedral stacking of protein may limit the efficacy to which cargo may be released. Although in silico and functional studies conducted in varying pH conditions [23] indirectly suggest that cargo release may be mediated in part by entry into the endosomolytic environment, the extent to which this occurs remains to be determined.

Thus far, HPK combined with the cargo summarized in Table 1 has produced bioparticles of similar size, shape, and targeting ability while delivering payloads with different functions. For example, small interfering RNAs (siRNAs) combined with HPK capsomeres form multivalent capsid-like structures or HerSi nano-nucleocapsids (NNCs) with gene silencing capability [22]. HerSi NNCs have exhibited a serum-stable protection of the siRNA in vitro and in vivo while showing the ability to target tumors and modulate target gene expression in both immunodeficient and immunocompetent tumor models of melanoma and triple-negative breast cancer [23]. The nucleic acid binding activity of HPK has also been exploited for the delivery of DNA-intercalating drugs. Specifically, the chemotherapeutic doxorubicin intercalates DNA as its tumoricidal mechanism of action [73,74], whereas this activity was also leveraged for non-covalent encapsulation in HPK nano-nucleocapsids, forming H3D or HerDox bioparticles [22]. Importantly, HerDox can deliver chemotoxicity to HER3+ tumor cells at a lower dose compared to untargeted doxorubicin but with similar efficacy and less off-target toxicity [22]. Other payloads bound to HPK include sulfonated gallium-metallated corroles (forming particles known as H3G or HerGa) that bear multi-functional benefits of tumor detection, diagnostics, and tumor toxicity based on the photophysical and tumoricidal properties of the corrole [22,67,68]. Likewise, sulfonated manganese-metallated corroles are tumor toxic and also bear paramagnetic activity that enable magnetic resonance (MR) detection with T1 shortening modulated by encapsulation in HPK particles (forming HerMn) [21]. The latter characteristic has facilitated the detection of tumor-selective corrole release in vitro and in vivo [21]. Similar to the net negative charge of nucleic acids imposed by the phosphate backbone, sulfonated corroles are unable to breach the cell membrane due to the negatively charged sulfonates, but can induce cytoxocity once entering the cytoplasmic space owing to the endosomolysis provided by HPK [22]. Importantly, the low to undetectable immunogenicity of these particles is likely due to the manner of particle formation whereby the exposed ligands distributed on the particle surface mask penton base epitopes and mimic the naturally occurring heregulin ligand [23,67].

### 4.3. Tumor Homing through Multivalent Binding with HER3

HPK uses the human epidermal growth factor receptor ErbB3 (HER3) to target bioparticles to tumor cells and induce tumor cell entry [18,19]. Numerous studies in recent years have highlighted an expanding role of HER3 in tumor progression [75,76,77,78,79,80,81,82,83,84,85,86,87], showing that its heightened cell surface density is associated with drug resistance (especially resistance to targeted inhibitors) and metastasis, including metastasis to the brain [75,76,81,88,89,90,91,92]. HER3 is a pseudokinase retaining an inactive kinase domain that heterodimerizes with and becomes phosphorylated by HER2 to activate the PI3K-Akt pathway which in turn can modulate cell growth and survival [93]. Because it lacks inherent kinase activity, targeting HER3 is not an efficient method to employ for signal inhibition [94,95]; however, increased HER3 on resistant tumor cells allows for missile-like tumor homing by nanocarriers such as HPK [21,22,23]. The HER3-specific binding ligand of HPK is derived from a minimal binding domain of neuregulin-1⍺1 [96] which, in comparison to neuregulin-1β1, appears to partially abrogate the activation of HER3 on tumor cells [22]. HER2+ breast cancer patients with acquired resistance to trastuzumab (Herceptin^®^) display a high density of HER3 expression on the cell surface [97]. HPK can facilitate the delivery of chemotherapeutics into HER3+ cells including those that resist trastuzumab and those that become metastatic [22]. Specifically, studies comparing trastuzumab-sensitive with trastuzumab-resistant HER2+ breast tumors show that while the HPK particles exhibit tumor-homing capability to both tumor types, particle accumulation is higher in the trastuzumab-resistant tumors concomitant with higher HER3 levels [22]. Additionally, secondary tumors formed after implantation of primary tumors displayed higher HER3 and concomitantly higher HPK particle accumulation compared with the primary tumors [22]. Importantly, a comparison of HPK to trastuzumab showed that trastuzumab sustains sparse localization and retention on the cell surface even at two hours after cell binding, whereas HPK induces rapid entry into the cell via the endocytic pathway after robust clustering at the cell surface [22].

**Figure 4:**
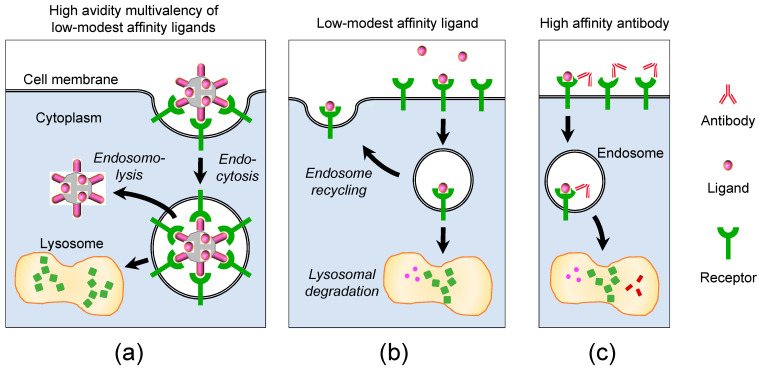
Multivalency and receptor binding. (**a**) Schematic representing multivalent binding of HPK bioparticle to multiple HER3 receptors, inducing endocytosis followed by evasion of lysosomal degradation through endosomal escape. (**b**) Schematic showing ligand–receptor interactions that may lead to the endolysosomal degradation pathway or recycling pathway. (**c**) Schematic of antibody-receptor binding suggesting that antibody internalization may be dependent on ligand-mediated co-uptake.

An important attribute of displaying the HER3 targeting ligand in the context of HPK is the avidity imposed by the natural oligomer formation of HPK. Whereas affinity reflects the binding strength of a receptor–ligand or a receptor–antibody interaction [98], avidity, also known as functional affinity, is the measure of the accumulated strength from the total affinities between all the binding interactions [98]. The receptor-binding proteins of viruses occur in multimers which can transform low-affinity single ligands into multivalent units with strong avidity. Likewise, the penton base portion of HPK drives its self-assembly into stable oligomers displaying multiple ligands from the same protein unit [23], thus forming a transductional targeting cluster with strong receptor-binding avidity (Figure 4a). As low to modest affinity ligands are less likely to undergo strong interaction with low receptor levels on non-tumor tissue, avidity through multivalency of low to modest affinity ligands promotes a preference for cells displaying high receptor levels [99,100], hence the proclivity for tumors. This contrasts with high affinity ligands or antibodies that may recognize even low to normal receptor levels and thus run the risk of delivering toxicity to off-target tissue, as described further below. This also contrasts with the phenomenon known as enhanced permeability and retention (EPR), which is mainly dictated by particle size and assumes that tumor vasculature is inherently leaky. Through accumulation in the tumor vasculature, EPR holds that certain cancer-targeting macromolecules over 40 kDa can deliver such therapeutics to the solid tumor [101]. EPR is dependent on the size and design of the macromolecule therapeutic, position of the vasculature within the tumor as well as its leakiness and the tumor location [102]. The main limitation of EPR is that while tumors grow, the vascular density and the transport between cells is decreased, and therefore, the EPR effect is decreased due to the decreased tumor vasculature accumulation, thus reducing passive delivery of therapeutics [103].

Another important aspect of the HPK design is that the targeting ligand cross-reacts with both mouse and human HER3 [23]. This allows mouse xenograft models of human cancer to be included as appropriate preclinical subjects for evaluating tumor homing in vivo as the particles can be assessed for targeting tumor cells that overexpress HER3 while avoiding normal levels inherent in the mouse despite the capacity to recognize both species of HER3 [22,23]. This contrasts with human species-specific targeting agents such as Herceptin® whose high affinity for human HER2 not only lacked sufficient recognition of the rodent receptor, but could also recognize even low levels of human HER2 [104]. It was not until the early clinical studies of Herceptin® that HER2 was identified on adult human heart tissue; its inhibition contributed to heart failure in clinical subjects, a finding that would not be evident in rodent testing [105]. A consideration of species specificity vs. cross-reactivity in the design of targeting ligands is often overlooked yet has important ramifications regarding the choice of appropriate preclinical models for rigorously evaluating tumor homing and the interpretations that can be made from data generated in such models.

Although HPK is now confirmed to be HER3-specific, its initial evaluation for targeting HER2+ tumor cells [19,20,67,69] was based on the propensity for neuregulins to interact with HER2+ tumors through the heterodimerization of HER2/ErbB2 with other ErbB receptor family members including HER3 [106], while HER2 lacks a ligand binding domain of its own [107,108,109]. The precise receptor specificity of HPK to the HER family members was initially not clearly identified and the majority of ligand–receptor binding studies were previously performed with the neuregulin1-beta isoform and not with the alpha isoform that comprises the receptor binding domain of HPK [110,111]. The specificity of HPK for HER3 has since been confirmed through several co-validating assays including receptor binding and competitive inhibition on immobilized HER3 in vitro and on HER3+ vs. HER3-deficient tumor cell lines; through the demonstration of preferential systemic accumulation in high- vs. low-HER3 tumors when both are present in mice; and by showing that the HPK nanobiologics exhibit increased binding and efficacy on trastuzumab- and lapatinib-resistant tumor cells concomitant with an increase in HER3 expression [22,23].

HER3-targeted antibody therapeutics are being tested in clinical trials but may have certain limitations. Antibody Drug Conjugates (ADCs) are composed of an antibody, in most cases a monoclonal antibody (mAb), bound to a drug usually through a chemical linker. Patritumab deruxtecan, produced by Daiichi Sankyo, in Phase 2 clinical trial is a topoisomerase I inhibitor conjugated to a IgG1 antibody that targets HER3+ breast cancer as well as EGFR-mutated non-small cell lung cancer [112]. This approach assumes that the inhibitor can be effectively trafficked to the nucleus after antibody binding to tumor cells. Although drug release in lysosomes is proposed and results show evidence of PARP cleavage and phosphorylation of DNA damage markers Chk1 and H2AX, it is unclear whether the payload deruxtecan localizes to the nucleus [112]. The therapeutic effect of targeted antibodies similar to patritumab deruxtecan may be due to antibody-dependent cellular cytotoxicity (ADCC) rather than any direct intracellular interactions presumed to take place after cell binding [113]. ADCC is an immune-mediated cytotoxic response that can be leveraged against a tumor cell through recognition between the FCgamma receptor R on effector cells and the Fc region of antibodies bound to cancer cells [114]. Other mAbs such as zenocutuzumab and seribantumab are both in Phase 2 clinical trials to treat NRG1-fusion+ solid tumors. Zenocutuzumab is an IgG1 antibody that targets both HER2 and HER3, while seribantumab is a human IgG2 mAB [115,116]. SI-B001 is an IgG2 antibody that is specific to both EGFR and HER3; it is also in Phase 2 clinical trials to treat head and neck, esophageal, lung and colorectal cancer [117]. HMBD-001 is in Phase 1 bladder cancer testing and Phase 2 trial to treat TNBC by targeting HER3+ solid tumors [118]. There are two other humanized IgG1 mAbs, ISU104 and AV-203, in clinical trials to treat head and neck squamous cell carcinoma as well as solid tumors [119,120]. The HER3 Antibody Radioisotope Conjugate (ARC) ^225^Ac-HER3-ARC, containing the radioisotope actinium-225 [121], and MP--EV20-ADC, which is conjugated to monomethyl auristatin F [122], are at the preclinical stage. In contrast to these HER3-targeted IgG-based antibodies, HPK can not only bind to HER3 using a minimal receptor binding domain, but also effectively penetrate through the endocytic pathway to deliver membrane-impermeable payloads (Figure 4a) including siRNA, sulfonated corroles, and nucleic acid-intercalated doxorubicin. ADCs may be limited in this regard as the immunorecognition of cell surface epitopes lacks the structure-induced conformational change that normally occurs when the natural ligand binds its receptor, as shown when neuregulin binds HER3 (Figure 4b,c) [123]. Likewise, antibody internalization may be dependent on co-uptake with the natural ligand, which in turn may lead to either a lysosomal degradation fate or recycling back to the cell surface (Figure 4b,c) [124].

## 5. Adenovirus Dodecahedron

The adenovirus dodecahedron comprises entirely self-assembled wild-type penton base proteins forming a polyhedron with twelve pentagonal faces (Figure 2c) [15,50]. The adenovirus dodecahedron was discovered by Erling Norrby in 1964, who isolated antigens of human cells infected by human adenovirus 3 [125]. He described the shape to include five or six pointed stars with a distance of 40 to 50 nm between the points. The most examined dodecahedron is from human adenovirus 3, and not all serotypes are capable of producing dodecahedra [15]. The dodecahedron shape differs based on angle of focus: when focusing on the vertices, it appears as an icosahedron; however, when focusing on the faces, the perceived shape of the dodecahedron is apparent [15].

Research and development applications of dodecahedra include their use for the delivery of nucleic acids and proteins [13,126,127]. Dodecahedra can be quickly internalized into a cell through multivalent wild-type penton base interaction with the cell membrane leading to membrane penetration for delivery of exogenous DNA and gene expression in vitro. A twenty-residue chimeric peptide comprising the fiber tail fused to a poly-lysine sequence was designed to electrostatically bind DNA cargo (through the polylysine) for attachment to the penton base (through the fiber tail) on the dodecahedron for in vitro DNA transport [126]. Although the approach of attaching DNA cargo on the outer surface of polyhedral carriers is not ideal for in vivo applications, dodecahedra prove the principle that the penton base (and those of different serotypes) is sufficient for breaching the cell membrane for the delivery of macromolecular cargo. This is further proven through the use of dodecahedra for delivering high-molecular-weight protein cargo [13]. Specifically, antibodies lack membrane-penetrating activity and thus cannot be delivered into the cytoplasm of cells. Human adenovirus 3 dodecahedra embellished with non-neutralizing monoclonal antibodies against the penton base successfully delivered the antibodies into human cells to access cytoplasmic targets which is otherwise not possible using the antibodies alone. Likewise, recombinant maltose binding protein produced as a fusion to three WW domains of the ubiquitin ligase Nedd4 could be successfully delivered into the cells by dodecahedra as the PPxY domains on dodecahedra serve as docking sites to bind with the recognized WW domains [15,128]. These examples support the sufficiency of the penton base alone to penetrate macromolecules into the cell interior.

## 6. Cell-Penetrating Peptides

Although the exact mechanism of cell entry for cell-penetrating peptides (CPPs) is largely speculative [129], CPPs have been explored for the penetration and delivery of cargo into cells. CPPs are short polypeptides composed of thirty to thirty-five amino acids or less and are usually composed of water-soluble and hydrophobic regions [130,131]. The proposed mechanisms can be divided into two main categories. The first is an energy-independent method of direct penetration through pore formation or through electrostatic forces in which the positively charged protein interacts with the negatively charged cell membrane with the goal of neutralizing a localized area of the cell surface enough to possibly gain entrance into the cell [132,133]. The second category relies on energy-dependent endocytosis and endosome escape [134].

The Cell Penetrating Peptide 2.0 Database (http://crdd.osdd.net/raghava/cppsite/, accessed on 8 February 2023) contains a total of 1855 entries including unique peptides. CPPs can be classified based on their origin (i.e., protein-derived, synthetic, or chimeric) [130], their conformation (cyclical or linear), and physicochemical character (cationic, amphipathic and hydrophobic) [130]. The TAT CPP is derived from the HIV Tat protein, and studies show TAT is responsible for nuclear localization of the viral genome after infection [135,136]. GALA peptides, named after the repeating Glu–Ala–Leu–Ala amino acid sequences, are thought to assume an alpha-helical shape and are stable at a pH of 6 [137]. The GALA peptide includes thirty amino acids and is long enough to traverse the cell membrane phospholipid bilayer [138]. Another CPP includes CADY, which is a combination of aromatic tryptophan and cationic arginine residues that may also adopt an amphipathic helical conformation thought to traverse the cell membrane [130,139].

A Phase I clinical trial completed in 2011 examined the CPP p28, or azurin, in regard to treating refractory tumors that express p53 [140]. Azurin is a 28-amino-acid cytotoxin derived from cupredoxin azurin emitted by Pseudomonas aeruginosa [141]. In the trial, none of the participants presented any dose-limiting toxicities (DLTs), nor did they develop an immunoglobulin G antibody immune response to the p28 peptide therapeutic, and one of the patients had a complete response at 139 weeks [140]. Another Phase I clinical trial with pediatric patients was completed to treat recurrent or progressive CNS tumors [142]; high p53 expression was found in half of the tissue samples after treatment, and although tolerated by the patients, no objective responses were observed [142]. These studies suggest that CPPs may be safe, though it remains to be confirmed whether any measurable therapeutic effect can be attributed to the intended mechanism of cell penetration as opposed to an ADCC-like effect.

CPPs present a potential means of therapeutic delivery in treating cancer; however, a more extensive body of robust and rigorous studies could benefit the majority of the field to validate the cellular impact CPPs are purported to effectuate mechanistically. This would necessitate multiple complementary and confirmatory experiments to prove that therapeutic delivery in fact occurs due to membrane penetration. VP22, derived from herpes simplex virus-1 (HSV-1), was previously explored as a means of delivering gene therapy to hepatomas [143]. Likewise, the Tat protein derived from the human immunodeficiency virus as well as the Antennapedia (Antp) homeobox protein have been explored based on reports of observed translocation across the cell membrane. These proteins have been observed to accumulate at the cell nucleus, although closer analysis has suggested that this observation may likely be an artefact of fixation [144]. This further emphasizes the need for the basic research community to consider multiple, robust, and rigorous confirmatory studies in assessing the biological activity of a cell-penetrating molecule for therapeutic delivery and efficacy.

## 7. Discussion and Future Perspectives

Examining targeted macromolecular transport into tumors through the lens of developing HPK has helped us identify essential features, barriers and limitations that can guide therapeutic delivery strategies. Tumor-targeting moieties derived from naturally occurring ligands may have an advantage over strategies using immunorecognition of cell surface epitopes given the conformational and rapid cellular response imposed by such ligands on receptor-mediated endocytosis, which in turn can facilitate robust entry of macromolecular cargo [18,22,68,72]. Multivalency of such ligands combined with strong avidity is likely to promote a preferential accumulation of nanocarriers into tumors expressing high receptor levels (in contrast to non-tumor tissue) and thus contribute to lower effective dosage and improved safety [22,23]. Membrane penetration is essential for therapeutic cargo to access intracellular targets, the majority of which are likely to be extra-endosomal (i.e., cytoplasmic, nuclear, etc.). Accordingly, insight into viral endosomolytic mechanisms may lead to improved design of macromolecular delivery vehicles, which in turn may contribute to more selective, lower, and safer therapeutic dosages as observed in the development and preclinical testing of HPK bioparticles [22,23,61,145]. The stability of the assembled delivery system in storage and in serum or blood further contributes to tumor-selective delivery of the therapeutic by avoiding premature release of the therapeutic before reaching the tumor, and the capsid-forming features of the penton base contribute to the high serum-stability of HPK bioparticles [22,23]. It remains to be seen whether this high stability may impose limitations on facile release of the therapeutic intracellularly, although mechanistic studies suggest that the low pH endosomal environment that facilitates endosomolysis may also contribute to cargo release [23]. The relatively low pharmacological dosage required to enable therapeutic effect on tumor growth may be indicative of sufficient cargo release inside tumors [22,24,69]; but a better understanding of these steps may provide further insight on HPK and penton base fundamental mechanisms that contribute to effective delivery of macromolecular cargo into a cell.

## 8. Conclusions

The focus on targeting cancer cells highlights the need to understand intracellular trafficking mechanisms, the ways in which these might impact effective therapeutic delivery, and to rigorously design sufficiently robust experiments to evaluate intracellular fate and to prove or disprove the intracellular delivery hypotheses. Entry into a cancer cell, successful transit through the endolysosomal pathway, and delivery of a therapeutic cargo with a functional output are three challenging feats to accomplish that must be considered for designing a macromolecule delivery system. The early infection stage components of the double-stranded DNA adenovirus may serve as effective tools in penetrating cells and delivering therapeutic cargo. The evolution of HPK and the dodecahedron support the efficiency of the adenovirus penton base for intracellular delivery. As the never-ending plight to cure disease continues, future innovations for therapeutic targeting may benefit further from nature’s already created macromolecular delivery machinery within the adenovirus or similar pathogens.

## Figures and Tables

**Table 1:** Overview of HPK-based nanobiologics.

**Name of Nanobiologic**	**Therapeutic Cargo** **(Type of Therapeutic)**	**Mechanism of Action**	**In Vitro Disease Model**	**In Vivo Disease Model**
HerGa/H3-G [21,67,68]	Gallium corrole (Small molecule)	Mitochondrial and cytoskeletal disruption; Fluorescence imaging	Human HER2+ breast tumor lines: BT-474, BT-474 TR *, SKBR3, SKBR3 TR *	Female NU/NU mice with BT474 or BT474 TR * xenografts; Particle dosage per injection, route: 0.004 mg/kg corrole dose, IV
HerMn [21]	Manganese corrole (small molecule)	Mitochondrial and cytoskeletal disruption; Paramagnetism; MR detection and MRI	Human HER2+ tumor lines: BT474, MDA-MB-435; Human HER2 low tumor sub-line of MDA-MB-231; Human cardiosphere-derived cells (CDCs)	Female NU/NU mice with MDA-MB-435 xenografts; Particle dosage per injection, route: 0.00022 mg/kg corrole dose, IV
HerDox/H3-D [22,69]	Doxorubicin (Chemotherapeutic)	DNA-intercalating agent	Human HER2+ breast tumor lines: BT-474, BT-474 TR *, SKBR3, SKBR3 TR *, JIMT-1	Female NU/NU mice with JIMT-1 xenografts; Particle dosage per injection, route: 0.02 mg/kg doxorubicin dose, IV
HerSi [23]	siRNA (nucleic acid)	RNA interference	Human HER3+ melanoma tumor lines: MDA-MB-435, MDA-MB-435-Br4	Female NU/NU mice with MDA-MB-435 xenografts; Particle dosage per injection, route: 0.087 mg/kg siRNA, IV; Female BALB/c mice with 4T1-Luc orthotopic implants; Particle dosage per injection, route: 0.087 mg/kg siRNA, IV

* TR, Trastuzumab-resistant.
